# Emerging Evidence and Critical Issues with the Use of Single-Drop Capillary Blood for the Measurement of Hemoglobin Concentration in Population-Level Anemia Surveys

**DOI:** 10.1016/j.advnut.2024.100290

**Published:** 2024-08-14

**Authors:** Crystal D Karakochuk, Omar Dary, Monica C Flores-Urrutia, Maria Nieves Garcia-Casal, Chika Hayashi, Maria Elena D Jefferds, Robert Johnston, Leila M Larson, Carine Mapango, Dora I Mazariegos Cordero, Denish Moorthy, Sorrel Namaste, Lisa M Rogers, Kuntal Saha, Sara Wuehler

**Affiliations:** 1 Food, Nutrition and Health, Faculty of Land and Food Systems, The University of British Columbia, Vancouver, Canada; 2 Bureau for Global Health, Maternal and Chid Health and Nutrition Office, USAID, Washington, D.C., United States; 3 Department of Nutrition and Food Safety, World Health Organization, Geneva, Switzerland; 4 Data & Analytics Section, Division of Data, Analytics, Planning and Monitoring, United Nations Children's Fund, New York, United States; 5 Nutrition Branch, Division of Nutrition, Physical Activity and Obesity, Centers for Disease Control and Prevention, Atlanta, GA, United States; 6 Department of Health Promotion, Education, and Behavior, Arnold School of Public Health, University of South Carolina, Columbia, SC, United States; 7 Nutrition and Micronutrient Department, Institute of Nutrition of Central America and Panamá, Guatemala City, Guatemala; 8 NuMERAL, RTI International, Washington, D.C., United States; 9 Program Operations Unit, Nutrition International, Ottawa, Ontario, Canada

**Keywords:** accuracy, anemia, blood specimen, capillary, hemoglobin, hemoglobinometer, measurement, precision, survey, venous

## Abstract

Accurate and precise measurement of hemoglobin concentration is critical for reliable estimations of anemia prevalence at the population level. When systematic and/or random error are introduced in hemoglobin measurement, estimates of anemia prevalence might be significantly erroneous and, hence, limit their usefulness. For decades, single-drop capillary blood has been the most common blood source used for the measurement of hemoglobin concentration in surveys, especially in low-income and middle-income countries. In this study, we highlight historical and emerging evidence that single-drop capillary blood introduces a high degree of random error (variability) to hemoglobin estimates, leading to less reliable estimates of anemia prevalence at the population level. At present, the best practice is to collect and use venous blood for measurement of hemoglobin with an automated hematology analyzer, following standard operating procedures and quality assurance measures. Where use of an automated analyzer is not possible, the analysis of venous blood in a point-of-care hemoglobinometer by trained phlebotomists or specimen collectors should be considered. A forthcoming systematic review will provide additional evidence on the accuracy and precision of single-drop capillary blood for hemoglobin assessment. In the meantime, we raise caution when using single-drop capillary blood for hemoglobin measurement as it can result in inaccurate hemoglobin estimates and less reliable anemia prevalence estimates.

**Statement of Significance**

For decades, single-drop capillary blood has been the most common blood source used for the measurement of hemoglobin concentration in surveys, especially in low-income and middle-income countries. However, emerging data have shown that single-drop capillary blood introduces a high degree of random error (variability) to hemoglobin estimates, leading to less reliable estimates of anemia prevalence at the population level. We caution against the use of single-drop capillary blood for hemoglobin measurement.

## Introduction

Hemoglobin is a protein in red blood cells that primarily functions to carry oxygen throughout the body to tissues [1]. Hemoglobin concentration is usually measured in a blood specimen and is used for the screening and diagnosis of anemia in both individuals and populations. Accurate and precise measurements of hemoglobin are critical for reliable estimations of anemia prevalence at the population level. When measurement error is introduced in the determination of hemoglobin concentration, estimates of anemia prevalence can be significantly influenced. Therefore, it is critical that the design, implementation, supervision, analysis, and reporting of hemoglobin concentration in surveys be carefully undertaken. This viewpoint article summarizes the collective discussion and considerations of the Technical Expert Advisory group on nutrition Monitoring (TEAM) working group for anemia, in the preparation of a WHO Technical Brief to operationalize best practice for hemoglobin measurement in population-level anemia surveys [2]. First, we summarize the types of blood specimens and common analytical methods for hemoglobin measurement and highlight the current standard operating procedures (SOPs) and the gaps within those SOPs. Next, we provide an overview of the common types of error observed in hemoglobin measurement (systematic and random error) and outline some of the published literature that highlight the critical issues in hemoglobin measurement. We conclude with a summary of the key knowledge gaps that have been identified by the TEAM working group and the current global actions that are underway to address some of the critical research gaps in hemoglobin measurement.

## Types of Blood Specimens and Common Analytical Methods for Hemoglobin Measurement

**FIGURE 1:**
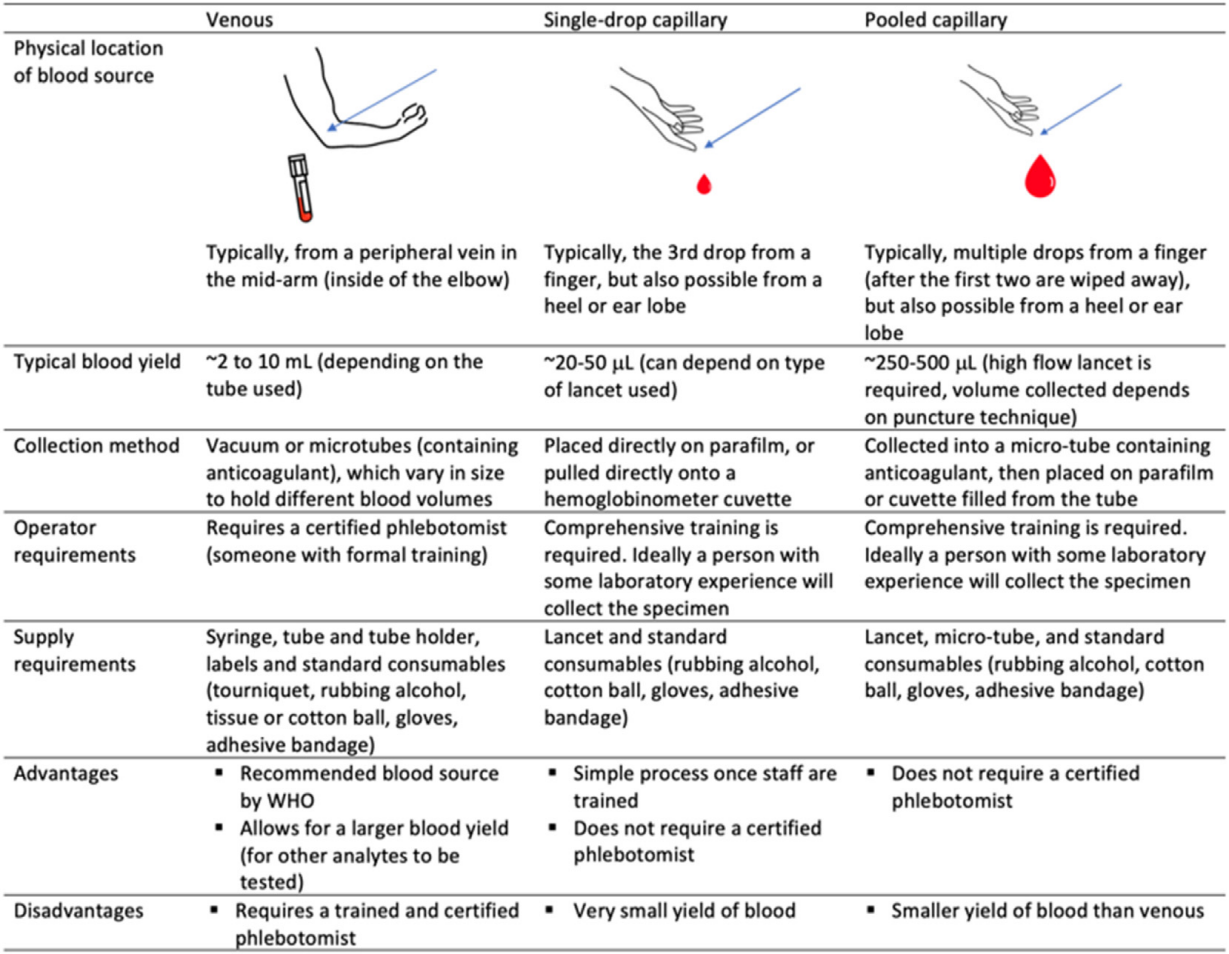
Overview of the blood sources often used for determination of hemoglobin concentration. WHO, World Health Organization.

Blood for hemoglobin concentration determination is obtained by either venous extraction or capillary prick via finger, heel, or earlobe (i.e., venous and capillary blood, respectively). Capillary blood can be collected as a single drop (typically the third drop, after the first 2 drops are wiped away) or a pooled specimen (multiple drops are collected in a tube with anticoagulant) (Figure 1). Several analytical devices are available to measure hemoglobin concentration, and detailed reviews of these analytical devices (including the key factors to consider when choosing which analytical method to use in an anemia survey, such as financial cost, resources, and cold chain) have been previously published [[2], [3], [4]]. The most common analytical devices are automated hematology analyzers and point-of-care hemoglobinometers. The WHO currently recommends venous blood as the preferred source, and measurement with an automated hematology analyzer, following SOPs and quality assurance measures [5].

## Current SOP for Hemoglobin Measurement

Several resources and SOP for hemoglobin measurement currently exist [2,[6], [7], [8]]. However, there are gaps within these SOP (Table 1 [2,[6], [7], [8]]) and in the current literature, such as a lack of defined criteria for minimum levels of acceptable random variation and systematic error. Despite these gaps, they remain useful guides to follow when planning and implementing a survey that measures hemoglobin. Current SOP for the measurement of hemoglobin has recently been summarized in a WHO Technical Brief [2], which describes the current best practices for hemoglobin measurement and includes additional information for field surveys that assess anemia prevalence at a population level.

**TABLE 1:** Existing resources and SOP for hemoglobin measurement.

Source	Topics covered
Centers for Disease Control and Prevention, World Health Organization, Nutrition International, UNICEF: Micronutrient Survey Manual & Toolkit 2022 [6]	The manual comprehensively covers 16 modules to design and implement a micronutrient survey, including biomarker selection and specimen handling (which briefly summarizes SOP for venous, capillary drop, and capillary pool specimen collection)
The DHS Program Biomarker Field Manual [7]	A training program for measuring and testing for biomarkers with use of capillary drop blood samples (finger or heel) for hemoglobin measurement with use of a HemoCue
WHO Guidelines on Drawing Blood: Best Practices in Phlebotomy 2010 [8]	Detailed technical guidance on the SOP for safe phlebotomy (for venous and capillary drop specimens) and details on materials required, complications, and other considerations. This may particularly be helpful when planning venous blood collection in population-level surveys
Best Practice for Hemoglobin Measurement in Population-level Anemia Surveys: Technical Brief [2]	Provides the most current guidance on best practices for hemoglobin measurement to those who are planning or implementing field surveys to assess population-level anemia prevalence; highlights the gaps in current knowledge for the collection of venous blood in the detailed protocol for collection of venous both for use with a hematology analyzer and point-of-care hemoglobinometer

Abbreviations: SOP, standard operating procedure.

## Types of Error in Hemoglobin Measurement

The reliable estimation of anemia prevalence in populations requires methods to determine the hemoglobin concentration that are accurate (i.e., that are similar to a gold standard or reference method, assuming that it is the true value), precise (i.e., low variability around the mean estimate values, and therefore good resolution to differentiate among values with similar hemoglobin concentration), and robust (i.e., the produced results will have the same certainty under different contexts, environments, and across populations, time points, and teams of technicians).

When ideal conditions for quality collection of a venous blood specimen, strong methodologic skills of the technicians, and optimal instrument performance are met, hemoglobin measurements can be accurate, precise, and robust [3,4,9]. When these conditions are not met, errors are introduced to the data and estimations of population-level anemia prevalence may be erroneous. This is particularly problematic when the population’s mean hemoglobin concentration is close to the cutoff for anemia diagnosis because anemia estimations are based on the proportion of individuals with hemoglobin measured below the determined thresholds. In other words, the magnitude of the error in anemia estimation also depends on the population’s mean hemoglobin value.

It is critical to note that there is no current global consensus of what magnitude of systematic error (method bias) and/or random error [limit of agreements (LOA)] are deemed acceptable in hemoglobin measurement for population-level surveys. This is a gap that is not clearly addressed in any current guidance or SOP. Table 1 summarizes existing resources that provide helpful and distinct information for hemoglobin measurement. However, all of them have 2 clear gaps in technical guidance: *1*) how to comprehensively assess hemoglobin data for quality (e.g., assessing total measurement error) in order to determine whether the data are accurate and precise (thus, reliable); and *2*) the minimum criteria of acceptable measurement error for population-level surveys. In individual-level diagnostic testing for anemia (such as in clinical laboratories), there are criteria proposed by some authors for acceptable variation, indicated as a target value of ±4% of single results of the individual hemoglobin concentration [10], which corresponds to ∼3 to 5 g/L difference between duplicate values in the clinically meaningful range of 80 to 130 g/L. Currently, there are no target values set for hemoglobin measurement error in population-level anemia estimates, and this is an area that requires global discussion and consensus.

## Common Types of Error Observed in Hemoglobin Measurement

Systematic error is a consistent or proportional difference between the observed and true values of a measure (e.g., an instrument that consistently measures hemoglobin concentrations as higher than they actually are). Systematic error can be used as an estimation of the accuracy of the method [11]. The magnitude of the error accounts for how far the experimental value is from the “true value.” The magnitude of systematic error can be constant or concentration dependent (e.g., if the magnitude of the error differs at low or high concentrations of hemoglobin). The mean difference of the test method results against the gold standard is the bias, and it can be calculated through the mean difference of the results of the method in use (test method) against those of the gold standard method or by using regression analysis between the results of the 2 methods [12]. There is potential for systematic error to be corrected or adjusted for, based on the difference of the hemoglobin value measured when compared with the hemoglobin values measured by gold standard methods or estimating the linear correlation of test and reference values over a range of hemoglobin concentrations. However, currently, there is no global consensus if this approach of adjusting systematic error is appropriate and, if so, under what circumstances. For hemoglobin measurement, the gold standard for instrumentation is spectrophotometric determination with the cyanmethemoglobin method. Most auto analyzers used by accredited clinical laboratories are based on this standard method and considered as an acceptable gold standard.

Random error refers to precision in the measurement. Random error is the variation of single results of the method around the mean predicted result, and it depends on numerous factors [3,4,9]: the intrinsic variability of the method, the heterogeneity of the analyte in the specimen, as well as the skills of the technician handling the specimens and using the method. The magnitude of the random error can only be estimated if replicate measurements are taken in the same sample or against results obtained with a reference method. Contrary to systematic error, random error or variability (due to its random nature) cannot be corrected for. Unless a high number of readings are performed and an mean calculated, the results of single readings may be highly erroneous. Random error can only be minimized through reducing the direct sources of variability. The first critical step is to identify what factors are causing the variability, which in itself can be very challenging. Ultimately, some degree of variation can be prevented, such as with following globally endorsed SOP for hemoglobin measurement [2,6] to reduce variation caused by improper technique or inappropriate blood collection protocols.

**FIGURE 2:**
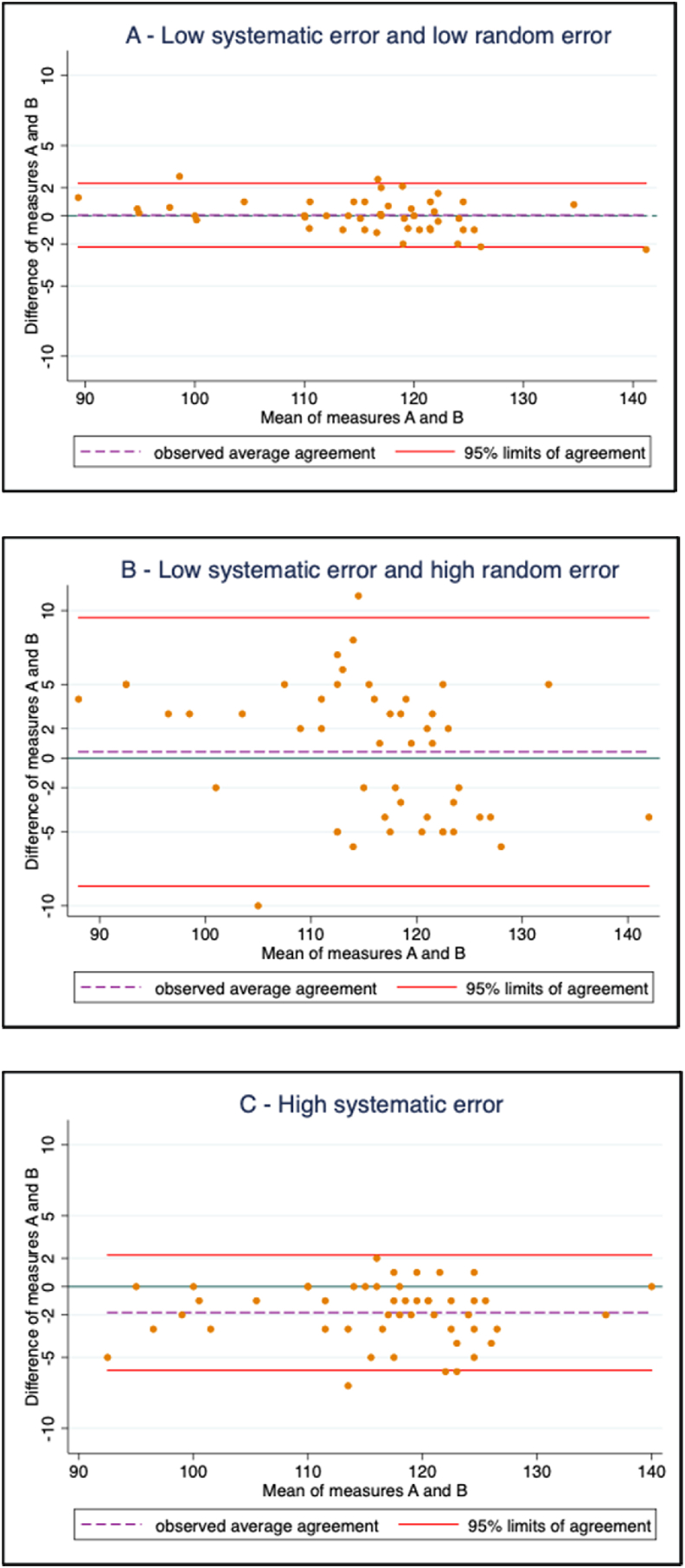
Examples of common error in hemoglobin measurement: (A) low systematic error and low random error (bias: 0.05 g/L; LOA: −2.2, 2.3 g/L); (B) low systematic error and high random error (bias: 0.4 g/L; LOA: −8.7, 9.5 g/L); and (C) high systematic error (bias: −1.8; LOA: −5.9, 2.2 g/L). Green horizontal line at y = 0 depicts no bias (perfect agreement between methods). Note: There is no global consensus on what specific criteria should be used here to identify the definitive presence of either random or systematic error; thus, the terms low and high error used in this figure are relative. LOA, limits of agreement.

It is also important to consider that systematic and random errors can occur simultaneously and it can be difficult to elucidate the causes of both sources of error. For visual interpretation of these types of error, Figure 2 uses simulated data to present examples of Bland–Altman plots depicting the bias (distance from the identity of “0”) and the LOA (represented as 95% CI from the mean) of 2 hemoglobin measurements. The figure illustrates 3 different scenarios of the combination of these 2 types of error. These graphs can be produced with statistical software and provide 1 approach to assess the reliability or quality of the data; however, this approach requires a substudy in field settings and comparison with a gold standard, which poses additional challenges. It is also important to assess the quality of the data before analysis and interpretation—this includes checking for potentially erroneous values and reporting critical information (e.g., assessment of and adjustments for elevation and smoking) when reporting population-level anemia survey data [2]. However, it is important to note that the data quality indicators are very limited in their ability to detect the reliability or quality of hemoglobin measurements alone.

## Studies Highlighting the Critical Issues Related to Hemoglobin Measurement

Previous studies have demonstrated differences in hemoglobin values or anemia prevalence estimates that are thought to be due to different blood specimens (single-drop or pooled capillary, or venous) [[13], [14], [15], [16], [17]], or analytical devices used (e.g., automated hematology analyzers compared with point-of-care hemoglobinometers) [[18], [19], [20], [21], [22], [23], [24]], as well as due to environmental conditions (e.g., settings of high humidity) [25]. Other external factors have also been shown to influence hemoglobin estimates in a variety of settings (e.g., hydration status, position during blood collection, sample loading time). Variation in hemoglobin estimates have also been detected among different models [25,26] and operators of hemoglobinometers [27]. Recent review articles commissioned by WHO have summarized these issues [2,3,9].

Studies have demonstrated that imprecision or random error, as well as systematic error, are critical issues in hemoglobin measurement, which can have substantial impact on estimates of population-level anemia prevalence. For example, Stevens et al. [28] systematically reviewed surveys pairs (conducted within 18 mo of each other) that included hemoglobin concentration measured in women of reproductive age and preschool-aged children. They found that surveys measuring hemoglobin with the HemoCue 301 showed higher hemoglobin concentrations than near-in-time surveys using the HemoCue 201+ in both women (difference of 5.8 g/L; 95% CI: 3.2, 8.3 g/L) and children (4.3 g/L; 95% CI: 1.4, 7.2 g/L). They concluded that these differences produced sizable shifts in estimated anemia prevalence that altered the severity classification of anemia as a public health problem. Thus, factors that influence hemoglobin measurement and introduce error must be identified and controlled for to generate reliable estimates of anemia prevalence.

## The Impact of High Random Error on Hemoglobin and Anemia Estimates

**FIGURE 3:**
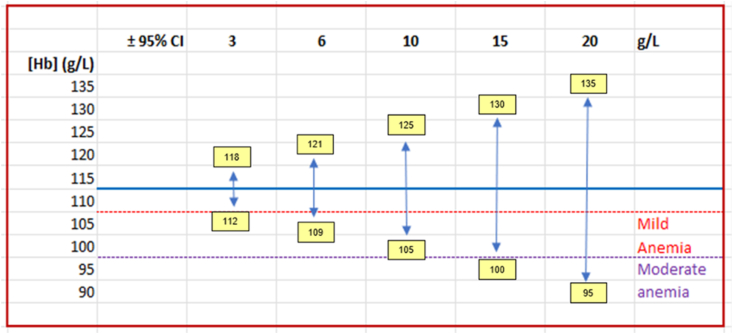
Example of the implications of random error on the precision of hemoglobin estimates in the diagnosis of anemia. With increasing random error (95% CI or LOA) from 3 to 20 g/L, the potential for misclassification of anemia also increases. This case presents an individual with a true mean hemoglobin concentration of 115 g/L. The diagnosis for mild anemia is 100–110 g/L and moderate anemia <100 g/L. The vertical blue lines include the range of possible values with single blood samples of this individual 95% of the time. Anemia would be confirmed in individuals if hemoglobin concentration was <110 g/L. If the method has a variation of 3 g/L or less (as typical in automated analyzers), this individual will almost always (within 95%) be identified without anemia (values between 112 and 118 g/L). However, if a drop of capillary blood of the same individual is analyzed in a point-of-care device, the variation can be as high as 20 g/L or more, and the results could be as low as 95 g/L (i.e., moderate anemia) and as high as 135 g/L. CI; confidence interval; LOA, limits of agreement.

**FIGURE 4:**
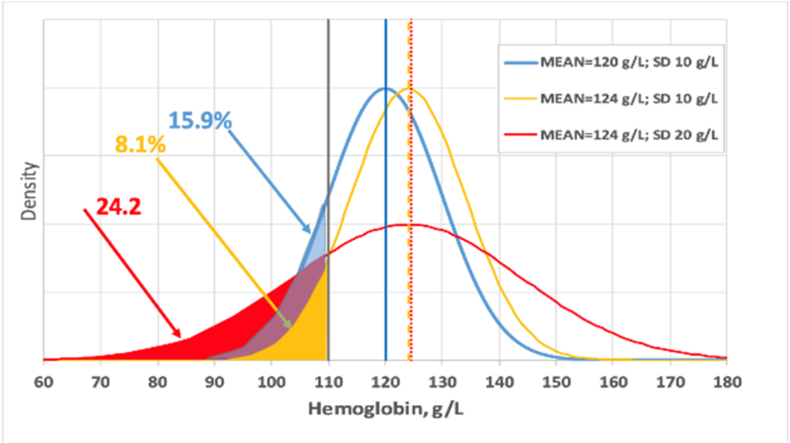
High random error (variability) can drastically influence anemia prevalence rates even when the mean hemoglobin values remain the same across comparisons. In this scenario, if we assume that the mean hemoglobin concentration of a population is 120 g/L with an SD of 10 g/L and that the threshold to diagnose anemia in that population is 110 g/L, then the prevalence of anemia is 15.9% (blue line). However, if the imprecision of the hemoglobin determination increases the SD to 20 g/L, the estimated anemia prevalence in the same population increases to 24.2% (red line). This problem continues even if the analytical method for the determination of hemoglobin has a positive systematic error (bias) of 4 g/L. Under the latter condition, if the SD is 10 g/L, the anemia prevalence is going to be underestimated at 8.1% (yellow line). Reproduced from reference [30] with permission. SD, standard deviation.

The issues of large variation in the hemoglobin concentration estimated using drops of capillary blood were highlighted in the literature decades ago; some researchers even recommended against their use [16,17]. However, the use of single-drop capillary blood to estimate hemoglobin concentration in point-of-care hemoglobinometers has continued to be widely used. Recently published articles indicate that random error is evident in hemoglobin estimates using single-drop capillary blood and substantially influences population-level anemia estimates [14,29]. Figure 3 illustrates the implications of random error on the precision of hemoglobin estimates in the diagnosis of anemia. Figure 4 illustrates scenarios by which the different degrees of random error could influence hemoglobin estimates and cause drastic differences in anemia prevalence rates [30]. In summary, random error in hemoglobin concentration can result in overestimation of the anemia prevalence.

Ultimately, inconsistencies across studies and populations (i.e., women and children) have warranted the need for a comprehensive review to systematically evaluate the degree of random error obtained when venous (the current gold standard), single-drop capillary, or pooled capillary blood is used for hemoglobin estimation for population-level anemia prevalence.

## Current Actions in Addressing the Critical Research Gaps in Hemoglobin Measurement

WHO recently updated the global recommendations to provide updated, evidence-informed recommendations on the hemoglobin cutoffs to define anemia and the best practices to measure it in individuals and populations as a strategy to improve the diagnosis of anemia [5]. WHO is commissioning a systematic review to assess the accuracy and precision of data collection and analytical methods for hemoglobin assessment in populations to systematically review the existing evidence. Further, in response to some of these critical issues in hemoglobin measurement, the United States Agency for International Development (USAID) convened the Hemoglobin Measurment (HEME) working group, a multi-institutional research initiative that brought together researchers and experienced practitioners working on hemoglobin measurement. Through this working group and the initiative of USAID, global research is currently ongoing in 6 countries (The Kingdom of Cambodia, the Federal Democratic Republic of Ethiopia, the Republic of Guatemala, the Lebanese Republic, the Federal Republic of Nigeria, and the United Republic of Tanzania) to identify the best procedures for hemoglobin measurement.

During the TEAM anemia working group discussions and revisions to the WHO Technical Brief [2], key knowledge gaps emerged and were discussed but not resolved, these include the following:

⁃ Can pooled capillary blood be collected under informed SOP in field settings so that potential measurement error can be reduced in population-level anemia assessment?
⁃ What is the maximum amount of time allowed between venous blood collection, cold chain storage, and hemoglobin analysis with use of the hematology analyzer (e.g., does venous blood require analysis within a specific amount of time after its collection)?
⁃ Does the presence of anticoagulants (e.g., EDTA, heparin) or the ratio (volume of anticoagulants to blood) affect hemoglobin measurement in blood?
⁃ Does the magnitude and source(s) of error differ by population (e.g., children compared with women of child bearing age, pregnant compared with nonpregnant women, and other groups)?
⁃ Is verification (or correction) of hemoglobinometers against hematology analyzers justified before their use to remove any potential individual systematic error (bias) of the device and, if so, in what circumstances?
⁃ What are the acceptable levels of systematic error (bias) and the random error (LOA), in hemoglobin measurement for estimating anemia prevalence in population surveys?

In conclusion, there is evidence that single-drop capillary blood introduces a high degree of random error to hemoglobin estimates and, thus, result in inaccurate estimates of population-level anemia prevalence. Therefore, at present, evidence suggests that the best practice is to collect and use venous blood for measurement of hemoglobin with an automated hematology analyzer, following SOPs as outlined in a recent WHO Technical Brief [2]. Where use of an automated analyzer is not possible, the analysis of venous blood in a point-of-care hemoglobinometer by trained phlebotomists or specimen collectors could be considered. Until more evidence is available, we raise caution when using single-drop capillary blood for hemoglobin measurement as it can result in inaccurate hemoglobin estimates and less reliable anemia prevalence estimates.

## Author contributions

The authors’ responsibilities were as follows – all authors: contributed to first draft of the manuscript and critically reviewed subsequent drafts and had final responsibility for the decision to submit for publication; and all authors: read and approved the final manuscript.

## Conflict of interest

The findings and conclusions in this report are those of the authors and do not necessarily reflect neither the official position or views of USAID or Centers for Disease Control and Prevention or the United States government nor other authors’ organizations. Use of trade names and commercial sources is for identification only and does not imply endorsement by any organization.

## Funding

This study is supported by a grant from the Bill & Melinda Gates Foundation to The World Health Organization.
